# Supplementary material for: SARS-CoV-2: Searching for the Missing Variants
Source: Viruses. 2022 Oct 26;14(11):2364. doi: 10.3390/v14112364 (PMC9697249; doi:10.3390/v14112364)
Supplement: Supplementary file 1 [file viruses-14-02364-s001.zip › viruses-1982154-supplementary.pdf]

```
Spike wt            MFVFLVLLPLVSSQCVNLTTRTQLPPAYTNSFTRGVYYPDKVFRSSVLHSTQDLFLPFFS  60
DELTA_              MFVFLVLLPLVSSQCVNLRTRTQLPPAYTNSFTRGVYYPDKVFRSSVLHSTQDLFLPFFS  60
omicronBA.1_        MFVFLVLLPLVSSQCVNLTTRTQLPPAYTNSFTRGVYYPDKVFRSSVLHSTQDLFLPFFS  60
omicronBA.2.75_     MFVFLVLLPLVSSQCVNLITRTQ---A-TNSFTRGVYYPDKVFRSSVLHSTQDLFLPFFS  56
omicronBA.2_        MFVFLVLLPLVSSQCVNLITRTQ---SYTNSFTRGVYYPDKVFRSSVLHSTQDLFLPFFS  57
omicronBA.2.12.1_   MFVFLVLLPLVSSQCVNLITRTQ---SYTNSFTRGVYYPDKVFRSSVLHSTQDLFLPFFS  57
omicronBA.4_        MFGFLVLLPLVSSQCVNLITRTQ---SYTNSFTRGVYYPDKVFRSSVLHSTQDLFLPFFS  57
omicronBA.5_        MFVFLVLLPLVSSQCVNLITRTQ---SYTNSFTRGVYYPDKVFRSSVLHSTQDLFLPFFS  57
                    ** *************** ****   : ********************************

Spike wt            NVTWFHAIHVSGTNGTKRFDNPVLPFNDGVYFASTEKSNIIRGWIFGTTLDSKTQSLLIV  120
DELTA_              NVTWFHAIHVSGTNGTKRFDNPVLPFNDGVYFASTEKSNIIRGWIFGTTLDSKTQSLLIV  120
omicronBA.1_        NVTWFHVI--SGTNGTKRFDNPVLPFNDGVYFASIEKSNIIRGWIFGTTLDSKTQSLLIV  118
omicronBA.2.75_     NVTWFHAIHVSGTNGTKRFDNPVLPFNDGVYFASTEKSNIIRGWIFGTTLDSKTQSLLIV  116
omicronBA.2_        NVTWFHAIHVSGTNGTKRFDNPVLPFNDGVYFASTEKSNIIRGWIFGTTLDSKTQSLLIV  117
omicronBA.2.12.1_   NVTWFHAIHVSGTNGTKRFDNPVLPFNDGVYFASTEKSNIIRGWIFGTTLDSKTQSLLIV  117
omicronBA.4_        NVTWFHAI--SGTNGTKRFDNPVLPFNDGVYFASTEKSNIIRGWIFGTTLDSKTQSLLIV  115
omicronBA.5_        NVTWFHAI--SGTNGIKRFDNPVLPFNDGVYFASTEKSNIIRGWIFGTTLDSKTQSLLIV  115
                    ******.*  ***** ****************** *************************

Spike wt            NNATNVVIKVCEFQFCNDPFLGVYYHKNNKSWMESEFRVYSSANNCTFEYVSQPFLMDLE  180
DELTA_              NNATNVVIKVCEFQFCNDPFLDVYYHKNNKSWMES--GVYSSANNCTFEYVSQPFLMDLE  178
omicronBA.1_        NNATNVVIKVCEFQFCNDPFLD---HKNNKSWMESEFRVYSSANNCTFEYVSQPFLMDLE  175
omicronBA.2.75_     NNATNVVIKVCEFQFCNDPFLDVYYHENNKSRMESELRVYSSANNCTFEYVSQPFLMDLE  176
omicronBA.2_        NNATNVVIKVCEFQFCNDPFLDVYYHKNNKSWMESEFRVYSSANNCTFEYVSQPFLMDLE  177
omicronBA.2.12.1_   NNATNVVIKVCEFQFCNDPFLDVYYHKNNKSWMESEFRVYSSANNCTFEYVSQPFLMDLE  177
omicronBA.4_        NNATNVVIKVCEFQFCNDPFLDVYYHKNNKSWMESEFRVYSSANNCTFEYVSQPFLMDLE  175
omicronBA.5_        NNATNVVIKVCEFQFCNDPFLDVYYHKNNKSWMESEFRVYSSANNCTFEYVSQPFLMDLE  175
                    *********************.   *:**** ***   **********************

Spike wt            GKQGNFKNLREFVFKNIDGYFKIYSKHTPINL--VRDLPQGFSALEPLVDLPIGINITRF  238
DELTA_              GKQGNFKNLREFVFKNIDGYFKIYSKHTPINL--VRDLPQGFSALEPLVDLPIGINITRF  236
omicronBA.1_        GKQGNFKNLREFVFKNIDGYFKIYSKHTPIIVREPEDLPQGFSALEPLVDLPIGINITRF  235
omicronBA.2.75_     GKQGNFKNLREFVFKNIDGYFKIYSKHTPVNL--GRDLPQGFSALEPLVDLPIGINITRF  234
omicronBA.2_        GKQGNFKNLREFVFKNIDGYFKIYSKHTPINL--GRDLPQGFSALEPLVDLPIGINITRF  235
omicronBA.2.12.1_   GKQGNFKNLREFVFKNIDGYFKIYSKHTPINL--GRDLPQGFSALEPLVDLPIGINITRF  235
omicronBA.4_        GKQGNFKNLREFVFKNIDGYFKIYSKHTPINL--GRDLPQGFSALEPLVDLPIGINITRF  233
omicronBA.5_        GKQGNFKNLREFVFKNIDGYFKIYSKHTPINL--GRDLPQGFSALEPLVDLPIGINITRF  233
                    *****************************: :   .************************

Spike wt            QTLLALHRSYLTPGDSSSGWTAGAAAYYVGYLQPRTFLLKYNENGTITDAVDCALDPLSE  298
DELTA_              QTLLALHRSYLTPGDSSSGWTAGAAAYYVGYLQPRTFLLKYNENGTITDAVDCALDPLSE  296
omicronBA.1_        QTLLALHRSYLTPGDSSSGWTAGAAAYYVGYLQPRTFLLKYNENGTITDAVDCALDPLSE  295
omicronBA.2.75_     QTLLALHRSYLTPGDSSSSWTAGAAAYYVGYLQPRTFLLKYNENGTITDAVDCALDPLSE  294
omicronBA.2_        QTLLALHRSYLTPGDSSSGWTAGAAAYYVGYLQPRTFLLKYNENGTITDAVDCALDPLSE  295
omicronBA.2.12.1_   QTLLALHRSYLTPGDSSSGWTAGAAAYYVGYLQPRTFLLKYNENGTITDAVDCALDPLSE  295
omicronBA.4_        QTLLALHRSYLTPGDSSSGWTAGAAAYYVGYLQPRTFLLKYNENGTITDAVDCALDPLSE  293
omicronBA.5_        QTLLALHRSYLTPGDSSSGWTAGAAAYYVGYLQPRTFLLKYNENGTITDAVDCALDPLSE  293
                    ******************.*****************************************

Spike wt            TKCTLKSFTVEKGIYQTSNFRVQPTESIVRFPNITNLCPFGEVFNATRFASVYAWNRKRI  358
DELTA_              TKCTLKSFTVEKGIYQTSNFRVQPTESIVRFPNITNLCPFGEVFNATRFASVYAWNRKRI  356
omicronBA.1_        TKCTLKSFTVEKGIYQTSNFRVQPTESIVRFPNITNLCPFDEVFNATRFASVYAWNRKRI  355
omicronBA.2.75_     TKCTLKSFTVEKGIYQTSNFRVQPTESIVRFPNITNLCPFHEVFNATRFASVYAWNRKRI  354
omicronBA.2_        TKCTLKSFTVEKGIYQTSNFRVQPTESIVRFPNITNLCPFDEVFNATRFASVYAWNRKRI  355
omicronBA.2.12.1_   TKCTLKSFTVEKGIYQTSNFRVQPTESIVRFPNITNLCPFDEVFNATRFASVYAWNRKRI  355
omicronBA.4_        TKCTLKSFTVEKGIYQTSNFRVQPTESIVRFPNITNLCPFDEVFNATRFASVYAWNRKRI  353
omicronBA.5_        TKCTLKSFTVEKGIYQTSNFRVQPTESIVRFPNITNLCPFDEVFNATRFASVYAWNRKRI  353
                    **************************************** *******************

Spike wt            SNCVADYSVLYNSASFSTFKCYGVSPTKLNDLCFTNVYADSFVIRGDEVRQIAPGQTGKI  418
DELTA_              SNCVADYSVLYNSASFSTFKCYGVSPTKLNDLCFTNVYADSFVIRGDEVRQIAPGQTGKI  416
omicronBA.1_        SNCVADYSVLYNLAPFFTFKCYGVSPTKLNDLCFTNVYADSFVIRGDEVRQIAPGQTGNI  415
omicronBA.2.75_     SNCVADYSVLYNFAPFFAFKCYGVSPTKLNDLCFTNVYADSFVIRGNEVSQIAPGQTGNI  414
omicronBA.2_        SNCVADYSVLYNFAPFFAFKCYGVSPTKLNDLCFTNVYADSFVIRGNEVSQIAPGQTGNI  415
omicronBA.2.12.1_   SNCVADYSVLYNFAPFFAFKCYGVSPTKLNDLCFTNVYADSFVIRGNEVSQIAPGQTGNI  415
omicronBA.4_        SNCVADYSVLYNFAPFFAFKCYGVSPTKLNDLCFTNVYADSFVIRGNEVSQIAPGQTGNI  413
omicronBA.5_        SNCVADYSVLYNFAPFFAFKCYGVSPTKLNDLCFTNVYADSFVIRGNEVSQIAPGQTGNI  413
                    ************ * * :****************************:** ********:*
```

```
Spike wt                   ADYNYKLPDDFTGCVIAWNSNNLDSKVGGNYNYLYRLFRKSNLKPFERDISTEIYQAGST  478
DELTA_                     ADYNYKLPDDFTGCVIAWNSNNLDSKVGGNYNYRYRLFRKSNLKPFERDISTEIYQAGSK  476
omicronBA.1_               ADYNYKLPDDFTGCVIAWNSNKLDSKVSGNYNYLYRLFRKSNLKPFERDISTEIYQAGNK  475
omicronBA.2.75_            ADYNYKLPDDFTGCVIAWNSNKLDSKVNGNYNYLYRLFRKSKLKPFERDISTEIYQAGNK  474
omicronBA.2_               ADYNYKLPDDFTGCVIAWNSNKLDSKVGGNYNYLYRLFRKSNLKPFERDISTEIYQAGNK  475
omicronBA.2.12.1_          ADYNYKLPDDFTGCVIAWNSNKLDSKVGGNYNYQYRLFRKSNLKPFERDISTEIYQAGNK  475
omicronBA.4_               ADYNYKLPDDFTGCVIAWNSNKLDSKVGGNYNYRYRLFRKSNLKPFERDISTEIYQAGNK  473
omicronBA.5_               ADYNYKLPDDFTGCVIAWNSNKLDSKVGGNYNYRYRLFRKSNLKPFERDISTEIYQAGNK  473
                           *********************:*****.***** *******:****************..

Spike wt                   PCNGVEGFNCYFPLQSYGFQPTNGVGYQPYRVVVLSFELLHAPATVCGPKKSTNLVKNKC  538
DELTA_                     PCNGVEGFNCYFPLQSYGFQPTNGVGYQPYRVVVLSFELLHAPATVCGPKKSTNLVKNKC  536
omicronBA.1_               PCNGVAGFNCYFPLRSYSFRPTYGVGHQPYRVVVLSFELLHAPATVCGPKKSTNLVKNKC  535
omicronBA.2.75_            PCNGVAGFNCYFPLQSYGFRPTYGVGHQPYRVVVLSFELLHAPATVCGPKKSTNLVKNKC  534
omicronBA.2_               PCNGVAGFNCYFPLRSYGFRPTYGVGHQPYRVVVLSFELLHAPATVCGPKKSTNLVKNKC  535
omicronBA.2.12.1_          PCNGVAGFNCYFPLRSYGFRPTYGVGHQPYRVVVLSFELLHAPATVCGPKKSTNLVKNKC  535
omicronBA.4_               PCNGVAGVNCYFPLQSYGFRPTYGVGHQPYRVVVLSFELLHAPATVCGPKKSTNLVKNKC  533
omicronBA.5_               PCNGVAGVNCYFPLQSYGFRPTYGVGHQPYRVVVLSFELLHAPATVCGPKKSTNLVKNKC  533
                           ***** *.******:**.*:** ***:*********************************

Spike wt                   VNFNFNGLTGTGVLTESNKKFLPFQQFGRDIADTTDAVRDPQTLEILDITPCSFGGVSVI  598
DELTA_                     VNFNFNGLTGTGVLTESNKKFLPFQQFGRDIADTTDAVRDPQTLEILDITPCSFGGVSVI  596
omicronBA.1_               VNFNFNGLKGTGVLTESNKKFLPFQQFGRDIADTTDAVRDPQTLEILDITPCSFGGVSVI  595
omicronBA.2.75_            VNFNFNGLTGTGVLTESNKKFLPFQQFGRDIADTTDAVRDPQTLEILDITPCSFGGVSVI  594
omicronBA.2_               VNFNFNGLTGTGVLTESNKKFLPFQQFGRDIADTTDAVRDPQTLEILDITPCSFGGVSVI  595
omicronBA.2.12.1_          VNFNFNGLTGTGVLTESNKKFLPFQQFGRDIADTTDAVRDPQTLEILDITPCSFGGVSVI  595
omicronBA.4_               VNFNFNGLTGTGVLTESNKKFLPFQQFGRDIADTTDAVRDPQTLEILDITPCSFGGVSVI  593
omicronBA.5_               VNFNFNGLTGTGVLTESNKKFLPFQQFGRDIADTTDAVRDPQTLEILDITPCSFGGVSVI  593
                           ********.***************************************************

Spike wt                   TPGTNTSNQVAVLYQDVNCTEVPVAIHADQLTPTWRVYSTGSNVFQTRAGCLIGAEHVNN  658
DELTA_                     TPGTNTSNQVAVLYQGVNCTEVPVAIHADQLTPTWRVYSTGSNVFQTRAGCLIGAEHVNN  656
omicronBA.1_               TPGTNTSNQVAVLYQGVNCTEVPVAIHADQLTPTWRVYSTGSNVFQTRAGCLIGAEYVNN  655
omicronBA.2.75_            TPGTNTSNQVAVLYQGVNCTEVPVAIHADQLTPTWRVYSTGSNVFQTRAGCLIGAEYVNN  654
omicronBA.2_               TPGTNTSNQVAVLYQGVNCTEVPVAIHADQLTPTWRVYSTGSNVFQTRAGCLIGAEYVNN  655
omicronBA.2.12.1_          TPGTNTSNQVAVLYQGVNCTEVPVAIHADQLTPTWRVYSTGSNVFQTRAGCLIGAEYVNN  655
omicronBA.4_               TPGTNTSNQVAVLYQGVNCTEVPVAIHADQLTPTWRVYSTGSNVFQTRAGCLIGAEYVNN  653
omicronBA.5_               TPGTNTSNQVAVLYQGVNCTEVPVAIHADQLTPTWRVYSTGSNVFQTRAGCLIGAEYVNN  653
                           ***************.****************************************:***

Spike wt                   SYECDIPIGAGICASYQTQTNSPRRARSVASQSIIAYTMSLGAENSVAYSNNSIAIPTNF  718
DELTA_                     SYECDIPIGAGICASYQTQTNSRRRARSVASQSIIAYTMSLGAENSVAYSNNSIAIPTNF  716
omicronBA.1_               SYECDIPIGAGICASYQTQTKSHRRARSVASQSIIAYTMSLGAENSVAYSNNSIAIPTNF  715
omicronBA.2.75_            SYECDIPIGAGICASYQTQTKSHRRARSVASQSIIAYTMSLGAENSVAYSNNSIAIPTNF  714
omicronBA.2_               SYECDIPIGAGICASYQTQTKSHRRARSVASQSIIAYTMSLGAENSVAYSNNSIAIPTNF  715
omicronBA.2.12.1_          SYECDIPIGAGICASYQTQTKSHRRARSVASQSIIAYTMSLGAENLVAYSNNSIAIPTNF  715
omicronBA.4_               SYECDIPIGAGICASYQTQTKSHRRARSVASQSIIAYTMSLGAENSVAYSNNSIAIPTNF  713
omicronBA.5_               SYECDIPIGAGICASYQTQTKSHRRARSVASQSIIAYTMSLGAENSVAYSNNSIAIPTNF  713
                           ********************:* ********************** **************

Spike wt                   TISVTTEILPVSMTKTSVDCTMYICGDSTECSNLLLQYGSFCTQLNRALTGIAVEQDKNT  778
DELTA_                     TISVTTEILPVSMTKTSVDCTMYICGDSTECSNLLLQYGSFCTQLNRALTGIAVEQDKNT  776
omicronBA.1_               TISVTTEILPVSMTKTSVDCTMYICGDSTECSNLLLQYGSFCTQLKRALTGIAVEQDKNT  775
omicronBA.2.75_            TISVTTEILPVSMTKTSVDCTMYICGDSTECSNLLLQYGSFCTQLKRALTGIAVEQDKNT  774
omicronBA.2_               TISVTTEILPVSMTKTSVDCTMYICGDSTECSNLLLQYGSFCTQLKRALTGIAVEQDKNT  775
omicronBA.2.12.1_          TISVTTEILPVSMTKTSVDCTMYICGDSTECSNLLLQYGSFCTQLKRALTGIAVEQDKNT  775
omicronBA.4_               TISVTTEILPVSMTKTSVDCTMYICGDSTECSNLLLQYGSFCTQLKRALTGIAVEQDKNT  773
omicronBA.5_               TISVTTEILPVSMTKTSVDCTMYICGDSTECSNLLLQYGSFCTQLKRALTGIAVEQDKNT  773
                           *********************************************:**************

Spike wt                   QEVFAQVKQIYKTPPIKDFGGFNFSQILPDPSKPSKRSFIEDLLFNKVTLADAGFIKQYG  838
DELTA_                     QEVFAQVKQIYKTPPIKDFGGFNFSQILPDPSKPSKRSFIEDLLFNKVTLADAGFIKQYG  836
omicronBA.1_               QEVFAQVKQIYKTPPIKYFGGFNFSQILPDPSKPSKRSFIEDLLFNKVTLADAGFIKQYG  835
omicronBA.2.75_            QEVFAQVKQIYKTPPIKYFGGFNFSQILPDPSKPSKRSFIEDLLFNKVTLADAGFIKQYG  834
omicronBA.2_               QEVFAQVKQIYKTPPIKYFGGFNFSQILPDPSKPSKRSFIEDLLFNKVTLADAGFIKQYG  835
omicronBA.2.12.1_          QEVFAQVKQIYKTPPIKYFGGFNFSQILPDPSKPSKRSFIEDLLFNKVTLADAGFIKQYG  835
omicronBA.4_               QEVFAQVKQIYKTPPIKYFGGFNFSQILPDPSKPSKRSFIEDLLFNKVTLADAGFIKQYG  833
omicronBA.5_               QEVFAQVKQIYKTPPIKYFGGFNFSQILPDPSKPSKRSFIEDLLFNKVTLADAGFIKQYG  833
                           ***************** ******************************************
```

```
Spike wt             DCLGDIAARDLICAQKFNGLTVLPPLLTDEMIAQYTSALLAGTITSGWTFGAGAALQIPF  898
DELTA_               DCLGDIAARDLICAQKFNGLTVLPPLLTDEMIAQYTSALLAGTITSGWTFGAGAALQIPF  896
omicronBA.1_         DCLGDIAARDLICAQKFKGLTVLPPLLTDEMIAQYTSALLAGTITSGWTFGAGAALQIPF  895
omicronBA.2.75_      DCLGDIAARDLICAQKFNGLTVLPPLLTDEMIAQYTSALLAGTITSGWTFGAGAALQIPF  894
omicronBA.2_         DCLGDIAARDLICAQKFNGLTVLPPLLTDEMIAQYTSALLAGTITSGWTFGAGAALQIPF  895
omicronBA.2.12.1_    DCLGDIAARDLICAQKFNGLTVLPPLLTDEMIAQYTSALLAGTITSGWTFGAGAALQIPF  895
omicronBA.4_         DCLGDIAARDLICAQKFNGLTVLPPLLTDEMIAQYTSALLAGTITSGWTFGAGAALQIPF  893
omicronBA.5_         DCLGDIAARDLICAQKFNGLTVLPPLLTDEMIAQYTSALLAGTITSGWTFGAGAALQIPF  893
                     *****************:******************************************

Spike wt             AMQMAYRFNGIGVTQNVLYENQKLIANQFNSAIGKIQDSLSSTASALGKLQDVVNQNAQA  958
DELTA_               AMQMAYRFNGIGVTQNVLYENQKLIANQFNSAIGKIQDSLSSTASALGKLQNVVNQNAQA  956
omicronBA.1_         AMQMAYRFNGIGVTQNVLYENQKLIANQFNSAIGKIQDSLSSTASALGKLQDVVNHNAQA  955
omicronBA.2.75_      AMQMAYRFNGIGVTQNVLYENQKLIANQFNSAIGKIQDSLSSTASALGKLQDVVNHNAQA  954
omicronBA.2_         AMQMAYRFNGIGVTQNVLYENQKLIANQFNSAIGKIQDSLSSTASALGKLQDVVNHNAQA  955
omicronBA.2.12.1_    AMQMAYRFNGIGVTQNVLYENQKLIANQFNSAIGKIQDSLSSTASALGKLQDVVNHNAQA  955
omicronBA.4_         AMQMAYRFNGIGVTQNVLYENQKLIANQFNSAIGKIQDSLSSTASALGKLQDVVNHNAQA  953
omicronBA.5_         AMQMAYRFNGIGVTQNVLYENQKLIANQFNSAIGKIQDSLSSTASALGKLQDVVNHNAQA  953
                     ***************************************************:***:****

Spike wt             LNTLVKQLSSNFGAISSVLNDILSRLDKVEAEVQIDRLITGRLQSLQTYVTQQLIRAAEI  1018
DELTA_               LNTLVKQLSSNFGAISSVLNDILSRLDKVEAEVQIDRLITGRLQSLQTYVTQQLIRAAEI  1016
omicronBA.1_         LNTLVKQLSSKFGAISSVLNDIFSRLDKVEAEVQIDRLITGRLQSLQTYVTQQLIRAAEI  1015
omicronBA.2.75_      LNTLVKQLSSKFGAISSVLNDILSRLDKVEAEVQIDRLITGRLQSLQTYVTQQLIRAAEI  1014
omicronBA.2_         LNTLVKQLSSKFGAISSVLNDILSRLDKVEAEVQIDRLITGRLQSLQTYVTQQLIRAAEI  1015
omicronBA.2.12.1_    LNTLVKQLSSKFGAISSVLNDILSRLDKVEAEVQIDRLITGRLQSLQTYVTQQLIRAAEI  1015
omicronBA.4_         LNTLVKQLSSKFGAISSVLNDILSRLDKVEAEVQIDRLITGRLQSLQTYVTQQLIRAAEI  1013
omicronBA.5_         LNTLVKQLSSKFGAISSVLNDILSRLDKVEAEVQIDRLITGRLQSLQTYVTQQLIRAAEI  1013
                     **********:***********:*************************************

Spike wt             RASANLAATKMSECVLGQSKRVDFCGKGYHLMSFPQSAPHGVVFLHVTYVPAQEKNFTTA  1078
DELTA_               RASANLAATKMSECVLGQSKRVDFCGKGYHLMSFPQSAPHGVVFLHVTYVPAQEKNFTTA  1076
omicronBA.1_         RASANLAATKMSECVLGQSKRVDFCGKGYHLMSFPQSAPHGVVFLHVTYVPAQEKNFTTA  1075
omicronBA.2.75_      RASANLAATKMSECVLGQSKRVDFCGKGYHLMSFPQSAPHGVVFLHVTYVPAQEKNFTTA  1074
omicronBA.2_         RASANLAATKMSECVLGQSKRVDFCGKGYHLMSFPQSAPHGVVFLHVTYVPAQEKNFTTA  1075
omicronBA.2.12.1_    RASANLAATKMSECVLGQSKRVDFCGKGYHLMSFPQSAPHGVVFLHVTYVPAQEKNFTTA  1075
omicronBA.4_         RASANLAATKMSECVLGQSKRVDFCGKGYHLMSFPQSAPHGVVFLHVTYVPAQEKNFTTA  1073
omicronBA.5_         RASANLAATKMSECVLGQSKRVDFCGKGYHLMSFPQSAPHGVVFLHVTYVPAQEKNFTTA  1073
                     ************************************************************

Spike wt             PAICHDGKAHFPREGVFVSNGTHWFVTQRNFYEPQIITTDNTFVSGNCDVVIGIVNNTVY  1138
DELTA_               PAICHDGKAHFPREGVFVSNGTHWFVTQRNFYEPQIITTDNTFVSGNCDVVIGIVNNTVY  1136
omicronBA.1_         PAICHDGKAHFPREGVFVSNGTHWFVTQRNFYEPQIITTDNTFVSGNCDVVIGIVNNTVY  1135
omicronBA.2.75_      PAICHDGKAHFPREGVFVSNGTHWFVTQRNFYEPQIITTDNTFVSGNCDVVIGIVNNTVY  1134
omicronBA.2_         PAICHDGKAHFPREGVFVSNGTHWFVTQRNFYEPQIITTDNTFVSGNCDVVIGIVNNTVY  1135
omicronBA.2.12.1_    PAICHDGKAHFPREGVFVSNGTHWFVTQRNFYEPQIITTDNTFVSGNCDVVIGIVNNTVY  1135
omicronBA.4_         PAICHDGKAHFPREGVFVSNGTHWFVTQRNFYEPQIITTDNTFVSGNCDVVIGIVNNTVY  1133
omicronBA.5_         PAICHDGKAHFPREGVFVSNGTHWFVTQRNFYEPQIITTDNTFVSGNCDVVIGIVNNTVY  1133
                     ************************************************************

Spike wt             DPLQPELDSFKEELDKYFKNHTSPDVDLGDISGINASVVNIQKEIDRLNEVAKNLNESLI  1198
DELTA_               DPLQPELDSFKEELDKYFKNHTSPDVDLGDISGINASVVNIQKEIDRLNEVAKNLNESLI  1196
omicronBA.1_         DPLQPELDSFKEELDKYFKNHTSPDVDLGDISGINASVVNIQKEIDRLNEVAKNLNESLI  1195
omicronBA.2.75_      DPLQPELDSFKEELDKYFKNHTSPDVDLGDISGINASVVNIQKEIDRLNEVAKNLNESLI  1194
omicronBA.2_         DPLQPELDSFKEELDKYFKNHTSPDVDLGDISGINASVVNIQKEIDRLNEVAKNLNESLI  1195
omicronBA.2.12.1_    DPLQPELDSFKEELDKYFKNHTSPDVDLGDISGINASVVNIQKEIDRLNEVAKNLNESLI  1195
omicronBA.4_         DPLQPELDSFKEELDKYFKNHTSPDVDLGDISGINASVVNIQKEIDRLNEVAKNLNESLI  1193
omicronBA.5_         DPLQPELDSFKEELDKYFKNHTSPDVDLGDISGINASVVNIQKEIDRLNEVAKNLNESLI  1193
                     ************************************************************

Spike wt             DLQELGKYEQYIKWPWYIWLGFIAGLIAIVMVTIMLCCMTSCCSCLKGCCSCGSCCKFDE  1258
DELTA_               DLQELGKYEQYIKWPWYIWLGFIAGLIAIVMVTIMLCCMTSCCSCLKGCCSCGSCCKFDE  1256
omicronBA.1_         DLQELGKYEQYIKWPWYIWLGFIAGLIAIVMVTIMLCCMTSCCSCLKGCCSCGSCCKFDE  1255
omicronBA.2.75_      DLQELGKYEQYIKWPWYIWLGFIAGLIAIVMVTIMLCCMTSCCSCLKGCCSCGSCCKFDE  1254
omicronBA.2_         DLQELGKYEQYIKWPWYIWLGFIAGLIAIVMVTIMLCCMTSCCSCLKGCCSCGSCCKFDE  1255
omicronBA.2.12.1_    DLQELGKYEQYIKWPWYIWLGFIAGLIAIVMVTIMLCCMTSCCSCLKGCCSCGSCCKFDE  1255
omicronBA.4_         DLQELGKYEQYIKWPWYIWLGFIAGLIAIVMVTIMLCCMTSCCSCLKGCCSCGSCCKFDE  1253
omicronBA.5_         DLQELGKYEQYIKWPWYIWLGFIAGLIAIVMVTIMLCCMTSCCSCLKGCCSCGSCCKFDE  1253
                     ************************************************************
```

```
Spike wt                DDSEPVLKGVKLHYT    1273
DELTA_                  DDSEPVLKGVKLHYT    1271
omicronBA.1_            DDSEPVLKGVKLHYT    1270
omicronBA.2.75_         DDSEPVLKGVKLHYT    1269
omicronBA.2_            DDSEPVLKGVKLHYT    1270
omicronBA.2.12.1_       DDSEPVLKGVKLHYT    1270
omicronBA.4_            DDSEPVLKGVKLHYT    1268
omicronBA.5_            DDSEPVLKGVKLHYT    1268
                        ***************
```

Figure S1. Sequences alignment. SARS-CoV-2 wild type spike protein and its variants delta and omicrons were aligned by using the Clustal Omega program (https://www.ebi.ac.uk/Tools/msa/clustalo/). For the alignment the default parameters were used (Dealign Input Sequences: no (false); Number of Combined Iterations: 0; Max Guide Tree Iterations: -1 (off); Max HMM Iterations: -1 (off); Use mBed-like clustering during subsequent iterations: yes (true); mBed-like Clustering Guide-tree: yes (true)).
